# A Fixed-Pattern Noise Correction Method Based on Gray Value Compensation for TDI CMOS Image Sensor

**DOI:** 10.3390/s150923496

**Published:** 2015-09-16

**Authors:** Zhenwang Liu, Jiangtao Xu, Xinlei Wang, Kaiming Nie, Weimin Jin

**Affiliations:** School of Electronic Information Engineering, Tianjin University, 92 Weijin Road, Nankai District, Tianjin 300072, China; E-Mails: liuzhenwang@tju.edu.cn (Z.L.); wangxinlei77@tju.edu.cn (X.W.); nkaiming@tju.edu.cn (K.N.); jwmtju@tju.edu.cn (W.J.)

**Keywords:** fixed-pattern noise (FPN), correction method, gray value compensation, CMOS image sensor (CIS), time-delay-integration (TDI)

## Abstract

In order to eliminate the fixed-pattern noise (FPN) in the output image of time-delay-integration CMOS image sensor (TDI-CIS), a FPN correction method based on gray value compensation is proposed. One hundred images are first captured under uniform illumination. Then, row FPN (RFPN) and column FPN (CFPN) are estimated based on the row-mean vector and column-mean vector of all collected images, respectively. Finally, RFPN are corrected by adding the estimated RFPN gray value to the original gray values of pixels in the corresponding row, and CFPN are corrected by subtracting the estimated CFPN gray value from the original gray values of pixels in the corresponding column. Experimental results based on a 128-stage TDI-CIS show that, after correcting the FPN in the image captured under uniform illumination with the proposed method, the standard-deviation of row-mean vector decreases from 5.6798 to 0.4214 LSB, and the standard-deviation of column-mean vector decreases from 15.2080 to 13.4623 LSB. Both kinds of FPN in the real images captured by TDI-CIS are eliminated effectively with the proposed method.

## 1. Introduction

Time-delay-integration (TDI) camera is a special kind of line-scan camera, which captures images through pixels arranged in an area array and works in line-scan mode. It is widely used for high-quality and low-noise imaging even under low illumination and at high scanning speed. TDI cameras play a key role in the remote push-broom sensing system to improve its low light level capability [1]. The TDI technique can be easily applied on charge-coupled device (CCD) which allows noiseless accumulation of signals in charge domain [2]. However, CCD requires high operating voltage and it is difficult to integrate signal processing circuits in it. CMOS technology is gaining attention in this application because of its low-power, low-cost and high integrated density [3]. However, it is more difficult to implement on-chip low-noise accumulation in analog domain and synchronous signal capturing of the image for all pixels in the same column with CMOS than with CCD. Therefore, the key technique in TDI-CMOS image sensor (TDI-CIS) is the signal accumulator.

Several reports on TDI-CIS have been published recently [4,5,6,7,8]. In our previous work, a 128-stage TDI-CIS with on-chip analog accumulator was proposed [9]. However, the parasitic resistors and capacitors of the accumulator can lead to row fixed-pattern noise (RFPN) when TDI-CIS works at high line rate. RFPN appears as obvious and periodic horizontal stripes in the output image. In addition, the structure of on-chip column-parallel readout circuit can lead to column fixed-pattern noise (CFPN) because of the technology fabrication deviation of TDI-CIS. CFPN appears as vertical stripes of different brightness in the output image. The stripes caused by RFPN and CFPN can lead to failure in data classification and incorrect retrievals of useful information. Therefore, both kinds of FPN degrade the output image quality seriously, thereby limiting the application of TDI-CIS. 

The RFPN caused by the specific circuit structure of the accumulator is a special kind of FPN and has not been reported in the literature. The CFPN of CIS is usually suppressed by on-chip circuit design [10]. However, the FPN of manufactured image sensors can only be eliminated by image postprocessing. Most existing destriping algorithms are designed for specific image sensors or data types—infrared imagery [11], hyperspectral/multispectral imagery [12,13], and moderate resolution imaging spectroradiometer (MODIS) [14,15]. The stripes to be removed in the literature are either horizontal or vertical, but horizontal stripes and vertical stripes interweave with each other in the output image of TDI-CIS. Therefore, the existing destriping algorithms are not suitable for TDI-CIS. 

In order to remove both kinds of stripes in the output image of TDI-CIS, a FPN correction method based on gray value compensation is proposed. In the proposed method, RFPN and CFPN are estimated and corrected based on the row-mean vector and column-mean vector of a large number of images captured under uniform illumination respectively. The remainder of this paper is organized as follows. Section 2 analyzes the sources and characteristics of FPN and establishes a noise model of TDI-CIS. Section 3 describes the proposed method. Section 4 presents the experimental results, and Section 5 provides the conclusions.

## 2. Analysis and Modeling of Sensor Noise

**Figure 1:**
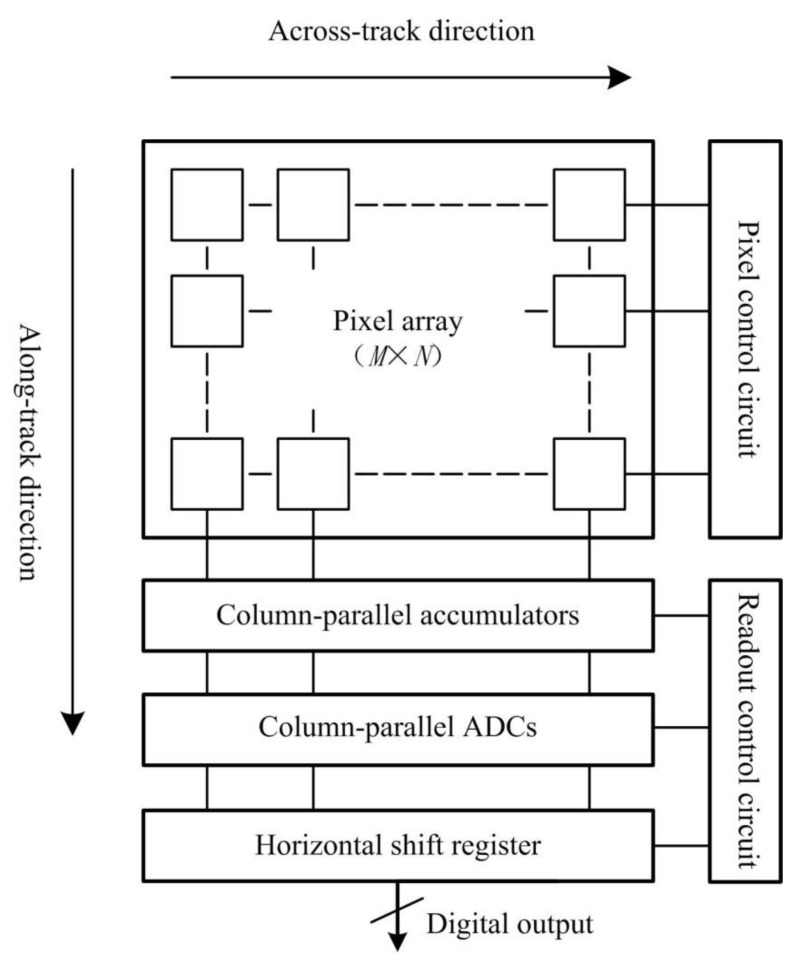
Block diagram of TDI-CIS.

TDI-CIS is mainly comprised of a pixel array, column-parallel accumulators and column-parallel analog-to-digital converters (ADCs). In Figure 1, a block diagram of TDI-CIS is depicted [16]. The scanning direction of TDI is called “along-track direction”, and the direction which is orthogonal to the along-track direction is called “across-track direction”. The noise of TDI-CIS can be simply divided into two types, one is FPN, and the other is random noise. In this paper, we only consider the correction of RFPN and CFPN.

### 2.1. Source and Analysis of RFPN

**Figure 2:**
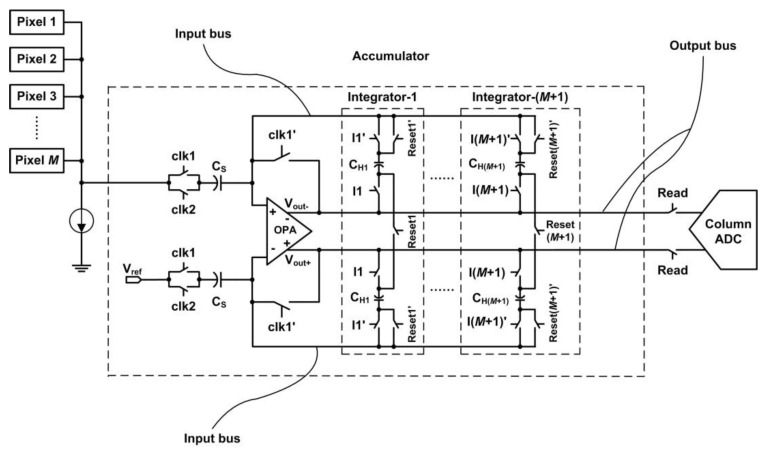
Circuit structure of the column-parallel accumulator.

As shown in Figure 1, the image sensor works in TDI mode with signal accumulation in analog domain. The circuit structure of the column-parallel accumulator is shown in Figure 2 [9]. The accumulator is mainly comprised of a fully-differential operational amplifier (OPA) and (*M* + 1) integrators. When TDI stage is *M*, the stage of the accumulator is (*M* + 1). The photoinduced charges are firstly stored in the integrators where they can be accumulated, then read out into the corresponding column ADC under the control of the sequential circuit.

**Figure 3:**
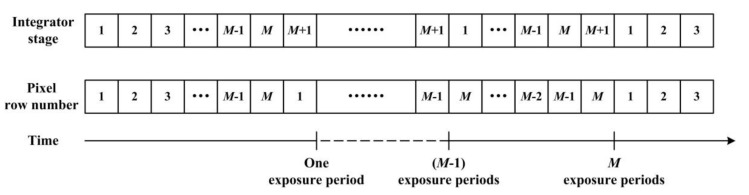
Correspondence between the integrator stage and the pixel row number in the pixel array.

TDI-CIS works in rolling-shutter readout mode in along-track direction with temporal oversampling rate of (M+1)/M, implementing synchronous signal capturing of the image for all *M* pixels in the same column [9,17]. The correspondence between the integrator stage and the pixel row number in the pixel array is shown in Figure 3. The charges stored in the (*M* + 1)-th-stage integrator, the *M-*th-stage integrator … and the 1st-stage integrator are read out in sequence and periodically under the control of the sequential circuit. Therefore, the gray value of the *P*-th row in the output image corresponds to the voltage signal read out from the (*M* + 2 - *P*)-th-stage integrator. The periodicity of the readout mode of the accumulator leads to the periodicity of the correspondence between the integrator stage and the pixel row number in the output image, and the period *T* is equal to the stage of the accumulator. Therefore, *T* can be expressed as:

(1) T=M+1

**Figure 4:**
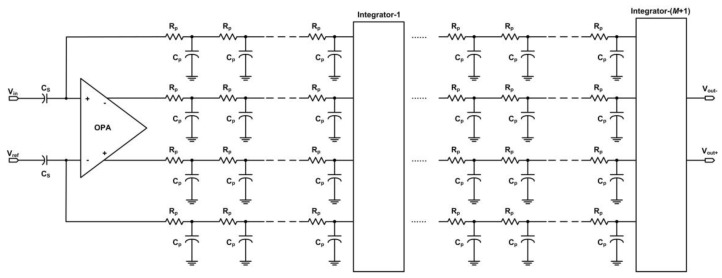
Parasitic resistors and capacitors in the buses of the accumulator.

As shown in Figure 2, the “input bus” and “output bus” of the accumulator are not ideal wires. There are parasitic resistors and capacitors in the layout of the buses, as shown in Figure 4. The parasitic parameters of an integrator vary with its corresponding length of buses. When TDI-CIS works at high line rate, the readout voltage values of different stages of integrator are different even under uniform illumination, and the readout voltage value of an integrator increases with increasing integrator stage. This is the reason why RFPN exists. In the output image, RFPN gray values of all pixels in the same row are equal, while RFPN gray value of a row changes periodically and increases with increasing row number in the along-track direction, and the period *T* satisfies Equation (1).

**Figure 5:**
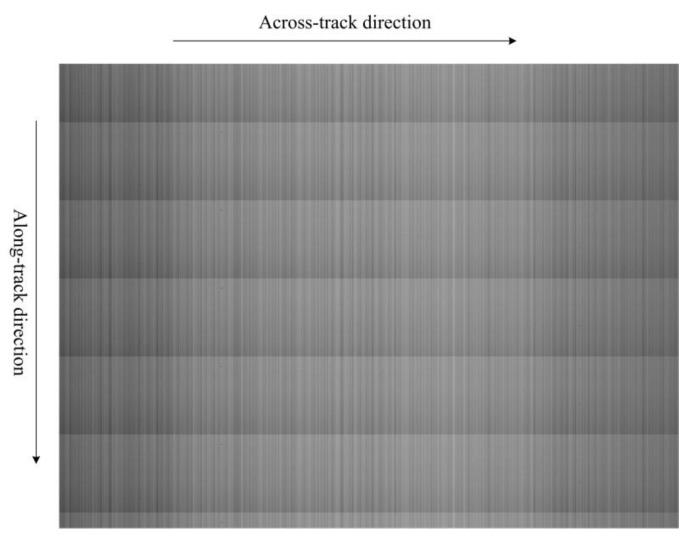
Original uniform-light image.

Figure 5 shows an original gray-level image captured by TDI-CIS under uniform illumination, which is called “uniform-light image”. The image size is 1024 × 768 pixels. Due to the existence of RFPN, the image brightness changes periodically and decreases with increasing row number in the along-track direction, leading to obvious horizontal stripes in the conjunctive areas of two adjacent periods.

### 2.2. Source and Analysis of CFPN

In Figure 1, there is a mismatch in both accumulators and ADCs of different columns because of the technology fabrication deviation of TDI-CIS. The mismatch leads to CFPN, which appears as vertical stripes of different brightness in the output image, just as shown in Figure 5.

In the output image, CFPN gray values of all pixels in the same column are equal, while CFPN gray values of pixels in different columns obey a zero-mean Gaussian distribution under the condition that the image size in across-track direction—*N*—is very large.

### 2.3. Noise Model of TDI-CIS

According to the analyses above, both RFPN and CFPN are a kind of spatial nonuniformity, and do not vary with illumination intensity. Both FPN and random noise are assumed to be additive noise, and they are uncorrelated. The FPN of a given manufactured TDI-CIS is assumed to be constant under the same working environment. The random noise obeys a zero-mean Gaussian distribution.

As shown in Figure 1, the size of the pixel array is M × N—*M* rows and *N* columns. The row number is defined as *i*, and the column number is defined as *j*. Assuming that the photoresponses of different pixels are uncorrelated, the gray value y(i,j) of pixel (i,j) in the output image can be expressed as:

(2) $$y(i,j)=x(i,j)-a(i)+b(j)+r(i,j)1\leq i\leq L,\;1\leq j\leq N$$

where *L* is the image size in along-track direction, x(i,j) is the ideal gray value of pixel (i,j), a(i) is the RFPN gray value of the *i-*th-row pixel, b(j) is the CFPN gray value of the *j-*th-column pixel, and r(i,j) is the sum of all kinds of noises except FPN. According to the analyses in Section 2.1, a(i) satisfies the following Equation:

(3) a(i)=a(i+T)

## 3. Method Description

According to the analyses in Section 2, the FPN correction method based on gray value compensation is proposed. Firstly sample data is acquired, and then RFPN and CFPN are estimated and corrected successively.

### 3.1. Sample Data Acquisition

In order to ensure effectiveness and accuracy of the estimation results of FPN, a large number of images should be captured with an image acquisition system. The image resolution of TDI-CIS is assumed to be 8-bit. Firstly *K* uniform-light images whose average gray values are all about 127—half-saturation—are captured under the same test environment [18]. Then the collected *K* images defined as $Y_{\text{1}},Y_{\text{2}},\ldots ,Y_{\text{K}}$ are selected as the sample data used for estimating FPN gray values. For any one image $Y_{\text{k}}$, Equation (2) can be expressed as:

(4) $$Y_{\text{k}}(i,j)=X_{\text{k}}(i,j)-a(i)+b(j)+r_{\text{k}}(i,j)1\leq k\leq K$$

### 3.2. Estimation and Correction of RFPN 

**Figure 6:**
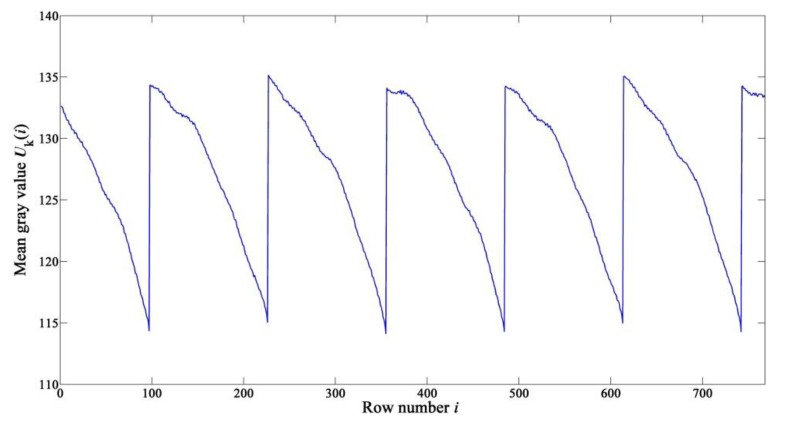
Mean gray value $U_{\text{k}}(i)$ *versus i*.

**Figure 7:**
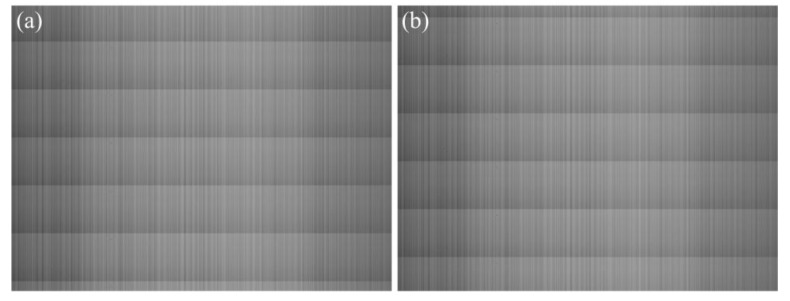
Two different uniform-light images. (**a**) Horizontal stripes in one kind of locations; (**b**) Horizontal stripes in another kind of locations.

The row-mean vector of $Y_{\text{k}}$ is defined as $U_{\text{k}}$, and the average gray value of the *i-*th-row pixels is defined as $U_{\text{k}}(i)$. As shown in Figure 6, due to the existence of RFPN, the curve of $U_{\text{k}}$ fluctuates strongly with periodic jumps in the locations of horizontal stripes. However, the locations of horizontal stripes in different images are not all the same, just as shown in Figure 7. Therefore, when calculating the row-mean vector of all collected images, the result obtained by averaging directly will be incorrect. In order to calculate the effective row-mean vector used for estimating RFPN, the locations of the first horizontal stripe in along-track direction of all collected images should be detected firstly, and this kind of operation is called “first line detection”.

The ratio of two adjacent elements in the row-mean vector of $Y_{\text{k}}$ is defined as $D_{\text{k}}(i)$, which can be expressed as:

(5) $$D_{\text{k}}(i)=\frac{U_{\text{k}}(i+1)}{U_{\text{k}}(i)}1\leq i\leq L-1$$

As shown in Figure 6, the average gray value of a row in which the horizontal stripes do not locate changes gradually and decreases with increasing row number in each period. Therefore, the locations where the jumps in the curve of $U_{\text{k}}$ take place can be easily got by threshold detection of $D_{\text{k}}(i)$ (such as larger than 1.15), *i.e*., the last row of every period can be detected. The row number of the last row of the first period is defined as $S_{\text{k}}$.

A new image $Y_{\text{C,k}}$ is constructed by cutting *t* periods from $Y_{\text{k}}$ starting from the ($S_{\text{k}}$ + 1)-th row. The image size of $Y_{\text{C,k}}$ is defined as $L_{\text{c}}\times N$, then the following equation can be deduced from Equation (1):

(6) $$L_{\text{c}}=t\times T=t\times (M+1)$$

Repeated the operations above on all collected images, *K* new images $Y_{\text{C,1}},Y_{\text{C,2}},\ldots ,Y_{\text{C,K}}$ can be constructed, in which the horizontal stripes are in the same locations. The row-mean vector of $Y_{\text{C,k}}$ is defined as $U_{\text{C,k}}$. Then the row-mean vector of all new images $Y_{\text{C,1}},Y_{\text{C,2}},\ldots ,Y_{\text{C,K}}$ can be expressed as:

(7) $$U_{\text{C}}=\frac{1}{K}\sum_{1}^{K}U_{\text{C,k}}$$

**Figure 8:**
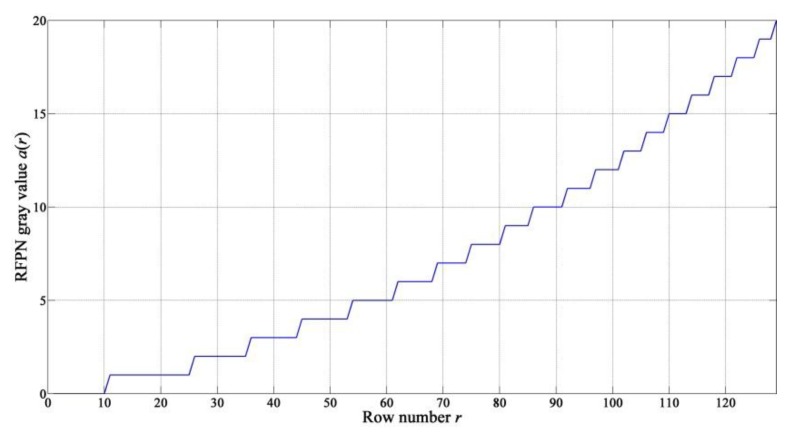
Estimated RFPN gray value a(r) *versus r* of one period.

$U_{\text{C}}$ is calculated by averaging based on multi-sample, so that the impacts of CFPN and random noise are eliminated. Therefore, a(m) ($1\leq m\leq L_{\text{c}}$) can be estimated based on the row-mean vector $U_{\text{C}}$. According to the analyses above, both a(m) and the elements in $U_{\text{C}}$ change periodically and therefore only one period of RFPN needs to be estimated. The estimated RFPN gray value is defined as a(r) (1≤r≤T), then a(r) can be estimated based on $U_{\text{C}}(r)$ (1≤r≤T).

a(r) increases with increasing row number, and it is the relative differences of a(r) between different rows that lead to RFPN, especially the horizontal stripes. Therefore, assuming that a(1)=0 will not reduce the effectiveness of RFPN correction. $U_{\text{C}}(1)$ minus $U_{\text{C}}(r)$ is exactly the estimated RFPN gray value of the *r-*th row. In consideration of the fact that the gray values are all integers, a(r) can be calculated according to the following Equation:

(8) $$a(r)=round(U_{\text{C}}(1)-U_{\text{C}}(r))2\leq r\leq M+1$$

where *round* is a MATLAB function which stands for rounding to the nearest integer. The estimated RFPN gray values of one period are shown in Figure 8.

According to Equation (2), RFPN can be corrected by adding the estimated RFPN gray value to the original gray values of pixels in the corresponding row. The operation of “first line detection” is required before RFPN correction, because the correspondence between a(i) in Equation (2) and a(r) in Equation (8) needs to be known firstly. In addition, in consideration of the fact that the range of gray values in 8-bit images is (0–255), constraints on the correction procedure should be set, as shown in the following Equation:

(9) $$z(i,j)=\{\begin{array}{l} y(i,j)+a(i)1\leq y(i,j)\leq 255-a(i)\\ y(i,j)y(i,j)=0,\text{ or }y(i,j)>255-a(i) \end{array}$$

where z(i,j) is the gray value of pixel (i,j) with RFPN corrected. When y(i,j)=0, the correction procedure is not carried out, because it cannot be judged whether the gray value y(i,j) satisfies Equation (2). 

### 3.3. Estimation and Correction of CFPN 

According to Equations (2) and (9), the gray value z(i,j) of pixel (i,j) in the image with RFPN corrected can be expressed as:

(10) $$z(i,j)=x(i,j)+b(j)+r(i,j)1\leq i\leq L,\;1\leq j\leq N$$

Carried out RFPN correction procedure for all collected images $Y_{\text{1}},Y_{\text{2}},\ldots ,Y_{\text{K}}$, *K* new images $Z_{\text{1}},Z_{\text{2}},\ldots ,Z_{\text{K}}$ can be got. The column-mean vector of $Z_{\text{k}}$ (1≤k≤K) is defined as $V_{\text{k}}$, then the column-mean vector of all new images $Z_{\text{1}},Z_{\text{2}},\ldots ,Z_{\text{K}}$ can be expressed as:

(11) $$V=\frac{1}{K}\sum_{1}^{K}V_{\text{k}}$$

**Figure 9:**
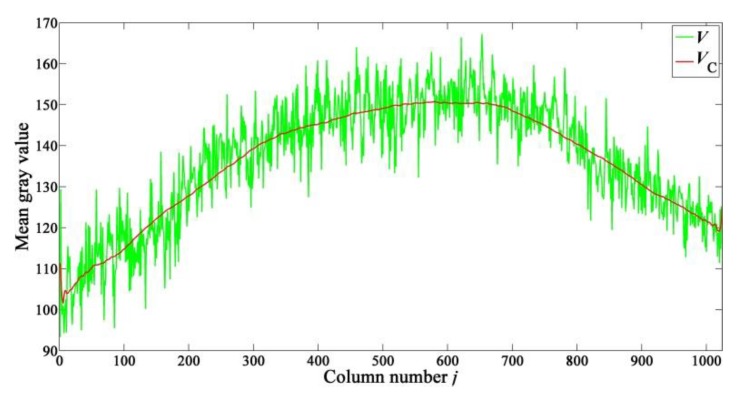
Curves of column-mean vectors V (shown by the green line) and $V_{\text{C}}$ (shown by the red line).

V is calculated by averaging based on multi-sample, so that the impacts of random noise are eliminated. Therefore, b(j) (1≤j≤N) can be estimated based on the column-mean vector V. Due to the existence of CFPN, the curve of V fluctuates strongly, as shown by the green line in Figure 9. In addition, the average gray values of central columns are larger than those of peripheral columns. A lens is used when capturing images. In order to clarify the reasons of this kind of shading, another group of sample data has been captured under dark condition. The column-mean vector of all collected images under dark condition is defined as $V_{\text{dark}}$. The experimental results show that, the distribution of $V_{\text{dark}}$ is nearly a flat curve. Therefore, the use of the lens is the only reason of the shading [19]. 

In order to estimate b(j) based on V, an ideal column-mean vector $V_{\text{C}}$ whose distribution is a nearly smooth curve should be constructed firstly, as shown by the red line in Figure 9. Then V minus $V_{\text{C}}$ is exactly the estimated CFPN vector.

$V_{\text{C}}$ can be constructed by taking the average of several adjacent elements in V—nearest-neighbor-averaging-filter (NNAF). The simplest NNAF is implemented by taking the average of two adjacent elements, but an ideal curve cannot be got by using the simplest NNAF. In order to construct $V_{\text{C}}$, a parametric NNAF expressed as the following Equation is designed:

(12) $$V_{\text{W}}(j)=\{\begin{array}{l} \frac{1}{2}(V(1)+V(2))j=1\\ \frac{1}{2j-1}\sum_{1}^{2j-1}V(n)2\leq j\leq W\\ \frac{1}{2W+1}\sum_{j-W}^{j+W}V(n)W+1\leq j\leq N-W\\ \frac{1}{2N-2j+1}\sum_{2j-N}^{N}V(n)N-W+1\leq j\leq N-1\\ \frac{1}{2}(V(N-1)+V(N))j=N \end{array}$$

where *W* is a variable, and $2\leq W\leq floor(\frac{N-1}{2})$; *floor* is a MATLAB function which stands for rounding toward negative infinity; $V_{\text{W}}$ is the column-mean vector constructed with the designed NNAF, and $V_{\text{W}}(j)$ is the average gray value of the *j-*th-column pixels. The maximum of *W* is defined as $W_{\max}$. In total, ($W_{\max}-1$) $V_{\text{W}}$s can be constructed when *W* ranges from 2 to $W_{\max}$.

The CFPN vector estimated based on $V_{\text{W}}$ is defined as $B_{\text{W}}$, and the average gray value of the *j-*th-column pixels is defined as $B_{\text{W}}(j)$. $B_{\text{W}}$ can be calculated according to the following Equation:

(13) $$B_{\text{W}}=floor(V-V_{\text{W}})$$

The average of all elements in $B_{\text{W}}$ is defined as $B_{\text{M,W}}$, and the absolute value of $B_{\text{M,W}}$ is defined as $B_{\text{MA,W}}$, then

(14) $$B_{\text{M,W}}=\frac{1}{N}\sum_{1}^{N}B_{\text{W}}(j)$$

(15) $$B_{\text{MA,W}}=\left|B_{\text{M,W}}\right|$$

**Figure 10:**
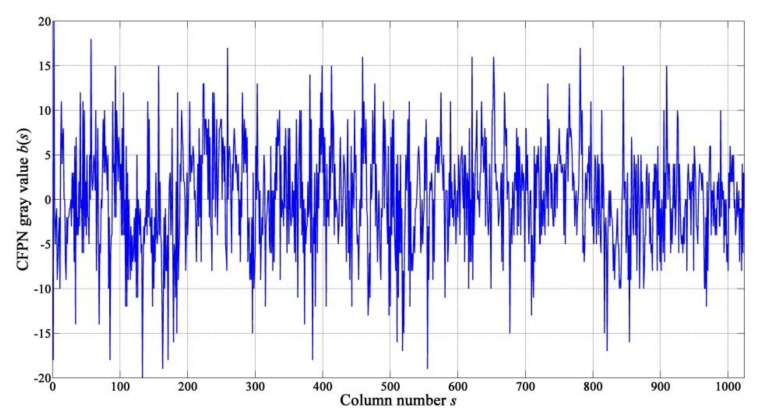
Estimated CFPN gray value b(s) *versus s*.

According to the analyses in Section 2.2, the elements in CFPN vector obey a zero-mean Gaussian distribution when *N* is very large, and therefore the average gray value of all elements in estimated CFPN vector should be zero or nearly zero. Then the $B_{\text{W}}$ whose corresponding $B_{\text{MA,W}}$ is minimal is the best estimation of CFPN vector. The best $B_{\text{W}}$ is defined as $B_{\text{S}}$, and the elements in $B_{\text{S}}$ are defined as b(s) (1≤s≤N), as shown in Figure 10.

According to Equation (10), CFPN can be corrected by subtracting the estimated CFPN gray value from the original gray values of pixels in the corresponding column, as shown in the following Equation:

(16) $$p(i,s)=\{\begin{array}{l} z(i,s)-b(s)b_{\max}\leq z(i,s)\leq 255+b_{\min}\\ z(i,s)z(i,s)<b_{\max},\text{ or }z(i,s)>255+b_{\min} \end{array}$$

where p(i,s) is the gray value of pixel (i,s) with CFPN corrected, $b_{\max}$ and $b_{\min}$ are the maximum and the minimum of b(s) (1≤s≤N) respectively. 

## 4. Experimental Results

In order to validate the effectiveness of the proposed method, a TDI CMOS imaging system based on a 128-stage TDI-CIS with on-chip analog accumulator is designed, and the following experiments are conducted. The TDI-CIS used in the experiments is the same device as that in our previous work [9]. Both RFPN and CFPN also exist in the previous work, but they had been corrected with the proposed method in this paper. This is the reason why FPN was not visible in [9]. The specifications of the TDI-CIS are listed in Table 1. 

**Table 1:** Specifications of the TDI-CIS.

Item	Description
Technology	0.18 µm CMOS
Power supply	1.8 V (Digital)/3.3 V (Analog)
Pixel array size	1024 columns × 128 rows
Pixel size	15 µm × 15 µm
Column ADC resolution	10-bit
Column ADC input range	1.6 V
Maximum line rate	3875 lines/s

**Figure 11:**
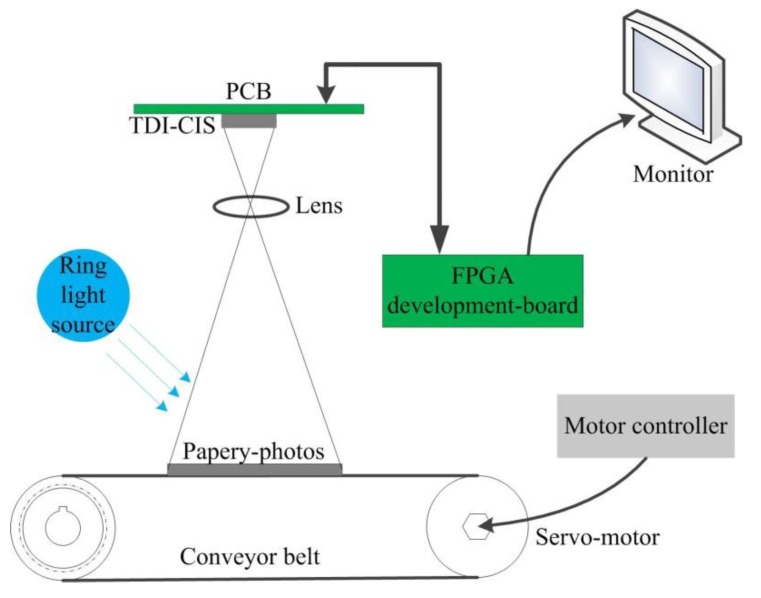
Schematic diagram of the TDI CMOS imaging system.

**Figure 12:**
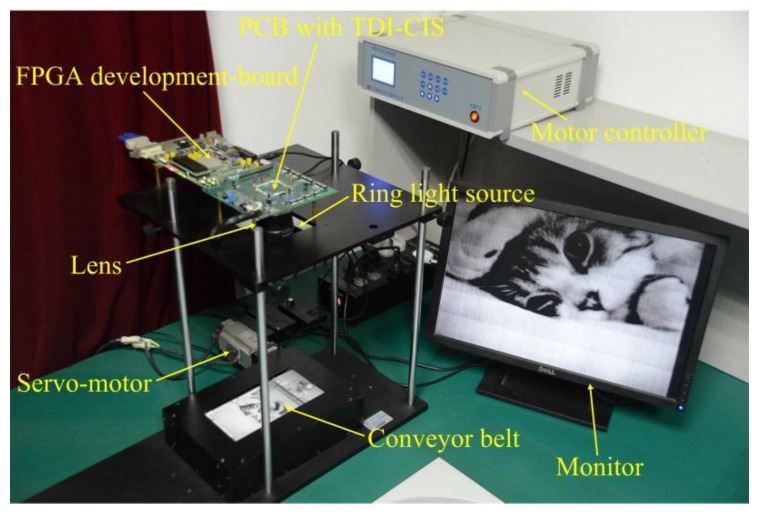
Photograph of the TDI CMOS imaging system.

The TDI CMOS imaging system is mainly comprised of seven parts—a ring light source used for providing uniform illumination, a lens, a conveyor belt on which original papery-photos are pasted, a servo-motor with controller used for scanning motion control, a printed-circuit-board (PCB) with the TDI-CIS, a field-programmable-gate-array (FPGA) development-board, and a monitor used for image display. The schematic diagram and photograph of the imaging system are shown in Figure 11 and Figure 12 respectively.

In the experiments, the TDI-CIS works at the line rate of 3875 lines/s. During sample data acquisition, a piece of white paper is used as the scene to be photographed to ensure that TDI-CIS works under uniform illumination. When papery-photos are selected as the scene to be photographed, the experiment is called “real-test experiment”, and the output image is called “real-test image”. In total, one hundred uniform-light images are collected as the sample data used for estimating FPN. The resolution of all output gray-level images is 8-bit. The experimental results are evaluated both subjectively and objectively. The subjective evaluation methods consist of “image visual effect” and “row-mean curve”. The objective evaluation indexes consist of “standard-deviation of row-mean vector (SDRMV)” and “standard-deviation of column-mean vector (SDCMV)”, both of which are given in the units of Least Significant Bit (LSB). The smaller the SDRMV is, the better the effectiveness of RFPN correction is. Similarly, the smaller the SDCMV is, the better the effectiveness of CFPN correction is. 

### 4.1. FPN Correction for Uniform-Light Image 

**Figure 13:**
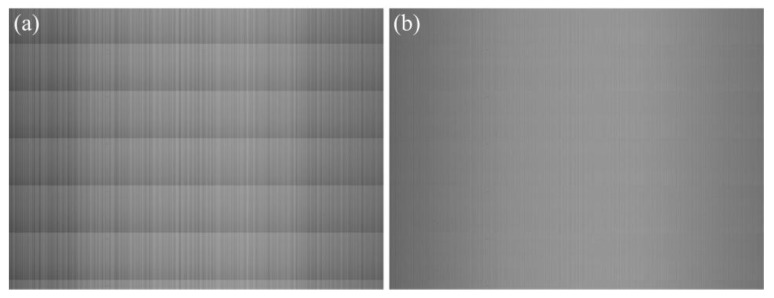
Comparison of visual effect of a uniform-light image. (**a**) Original image; (**b**) Corrected image.

The comparison of visual effect of a uniform-light image is shown in Figure 13. The image size is 1024×768 pixels. After correcting the FPN with the proposed method, the brightness of the corrected image is almost uniform in along-track direction, and the vertical stripes in across-track direction are significantly alleviated.

The comparison of objective evaluation indexes of the uniform-light image in Figure 13 is listed in Table 2. According to the analyses in Section 3.3, the use of the lens leads to that the average gray values of central columns are larger than those of peripheral columns, which is known as lens shading, as shown by the green line in Figure 9. The lens shading correction is not included in our method. This is the reason why both the SDCMVs of the original image and the corrected image in Table 2 are very large. As shown in Table 2, the SDRMV decreases from 5.6798 to 0.4214 LSB, and the SDCMV decreases from 15.2080 to 13.4623 LSB. The experimental results indicate that both RFPN and CFPN have been eliminated effectively.

**Table 2:** Comparison of objective evaluation indexes of the uniform-light image.

Image	SDRMV (LSB)	SDCMV (LSB)
Original image	5.6798	15.2080
Corrected image	0.4214	13.4623

### 4.2. FPN Correction for Real-Test Image 

**Figure 14:**
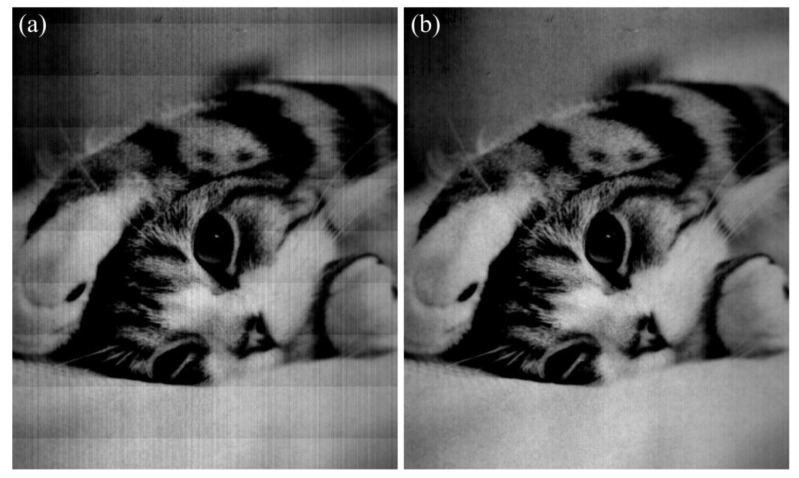
Comparison of visual effect of a real-test image. (**a**) Original image; (**b**) Corrected image.

The comparison of visual effect of a real-test image is shown in Figure 14. Due to the existence of FPN, there are obvious horizontal stripes in the conjunctive areas of two adjacent periods in the along-track direction, and vertical stripes of different brightness in across-track direction. After correcting the FPN with the proposed method, both kinds of stripes are removed. 

**Figure 15:**
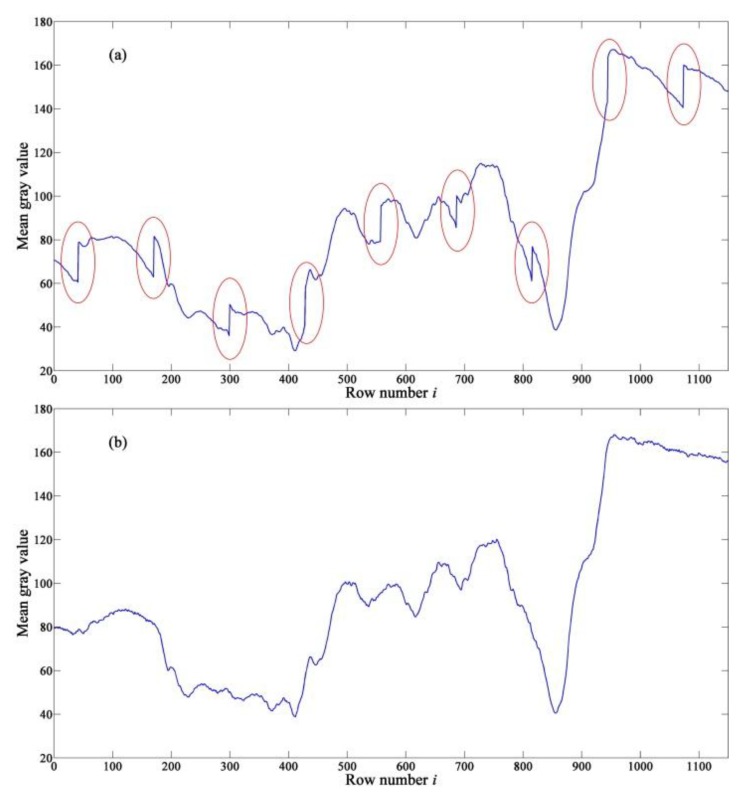
Comparison of row-mean curve of the real-test image in Figure 14. (**a**) Original curve; (**b**) Corrected curve.

The comparison of row-mean curve of the real-test image in Figure 14 is shown in Figure 15, where the curve of the original image is called “original curve”, and the curve of the corrected image is called “corrected curve”. The original curve fluctuates strongly with periodic jumps marked with ellipses in Figure 15a. According to Equation (1), the period of a 128-stage TDI-CIS is 129 rows. After correcting the FPN with the proposed method, the jumps disappear completely, and therefore the corrected curve is smoother than the original curve. In addition, both the form and change trend of the corrected curve are consistent with those of the original curve, demonstrating that the proposed method is able to correct FPN with useful information content of the original image well preserved.

**Figure 16:**
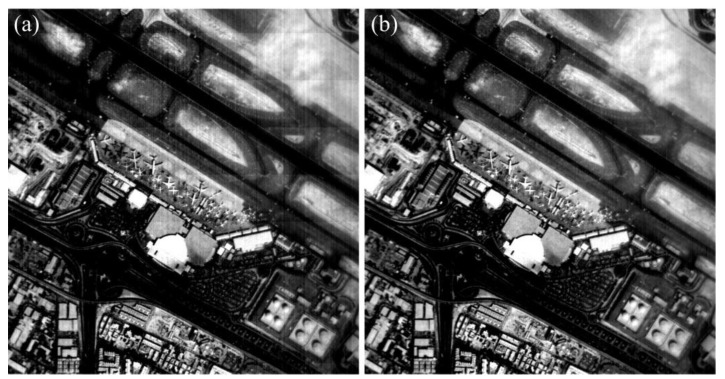
Comparison of visual effect of the Dubai International Airport. (**a**) Original image; (**b**) Corrected image.

**Figure 17:**
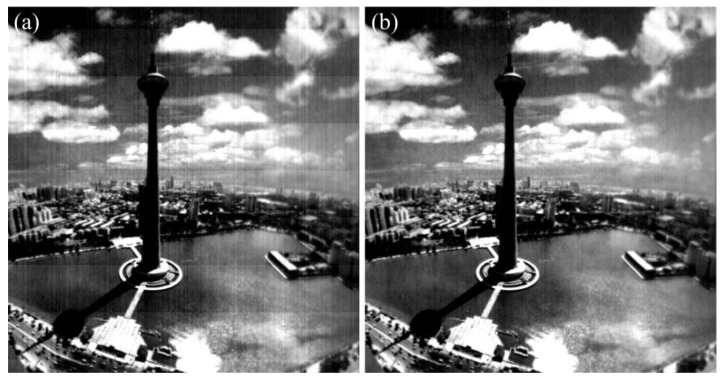
Comparison of visual effect of the Tientsin Tower. (**a**) Original image; (**b**) Corrected image.

**Figure 18:**
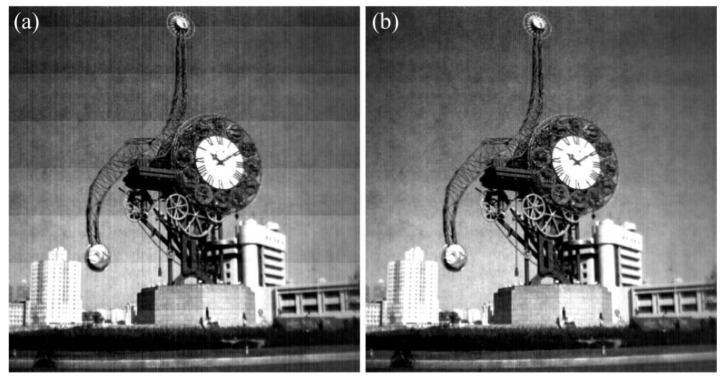
Comparison of visual effect of the Century Clock of Tianjin. (**a**) Original image; (**b**) Corrected image.

**Figure 19:**
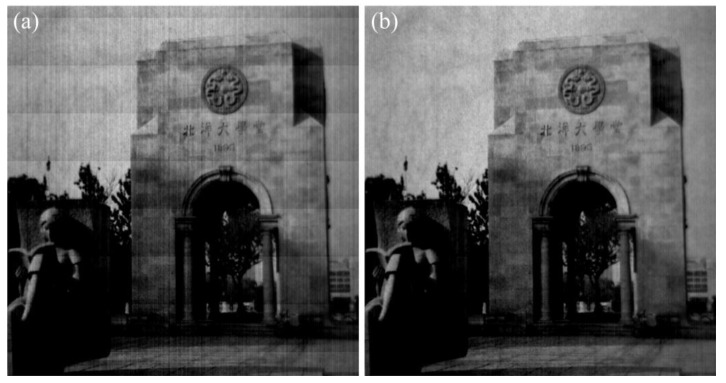
Comparison of visual effect of the Peiyang University Memorial. (**a**) Original image; (**b**) Corrected image.

In order to validate the robustness of the proposed method, the real-test experiment was conducted twenty times with the imaging system in Figure 12, and some of the experimental results are shown in Figure 16, Figure 17, Figure 18 and Figure 19. The stripes in different kinds of papery-photos are removed after correcting the FPN with the proposed method. 

### 4.3. Running Time 

In the experiments, both FPN estimation and FPN correction are accomplished in MATLAB R2014a on a personal computer with an Intel Core 2 CPU at 2.93-GHz and 2-GB memory. Before FPN correction, the FPN gray values are estimated in advance. The running time of the FPN correction procedure in different figures is listed in Table 3. All the running times are given in the units of Seconds. It can be seen that the FPN correction procedure is very fast.

**Table 3:** Running time of the FPN correction procedure in different figures.

Figure	Running Time (s)
Figure 13	0.392568
Figure 14	0.431741
Figure 16	0.391541
Figure 17	0.370390
Figure 18	0.361930
Figure 19	0.373811

## 5. Conclusions

In this paper, the sources of FPN and noise characteristics of TDI-CIS were analyzed, and then a FPN correction method based on gray value compensation was proposed. Experimental results based on a 128-stage TDI-CIS show that, both RFPN and CFPN in the output image are eliminated effectively and both image visual effect and objective evaluation indexes are improved; finally, the image quality becomes better. The proposed method is suitable for TDI-CIS, and is able to correct FPN with useful information content of the original image well preserved.

## References

[1] Farrier M.G., Dyck R.H. (1980). A Large Area TDI Image Sensor for Low Light Level Imaging. IEEE J. Solid-State Circuits.

[2] Wong H.-S., Yao Y.L., Schlig E.S. (1992). TDI charge-coupled devices: Design and applications. IBM J. Res. Dev..

[3] El Gamal A., Eltoukhy H. (2005). CMOS image sensors. IEEE Circuits Devices Mag..

[4] Lepage G., Dantes D., Diels W. CMOS long linear array for space application. Proceedings of the SPIE Sensors, Cameras, and Systems for Scientific/Industrial Applications VII.

[5] Kim C.B., Kim B.-H., Lee Y.S., Jung H., Lee H.C. Smart CMOS Charge Transfer Readout Circuit for Time Delay and Integration Arrays. Proceedings of the IEEE 2006 Custom Integrated Circuits Conference (CICC).

[6] Yu H., Qian X., Chen S., Low K.S. A Time-Delay-Integration CMOS image sensor with pipelined charge transfer architecture. Proceedings of the 2012 IEEE International Symposium Circuits and Systems (ISCAS).

[7] Chang J.-H., Cheng K.-W., Hsieh C.-C., Chang W.-H., Tsai H.-H., Chiu C.-F. Linear CMOS image sensor with time-delay integration and interlaced super-resolution pixel. Proceedings of the 2012 IEEE Sensors.

[8] Nie K., Yao S., Xu J., Gao J. (2014). Thirty Two-Stage CMOS TDI Image Sensor With On-Chip Analog Accumulator. IEEE Trans. Very Larg. Scale Integr. (VLSI) Syst..

[9] Nie K., Yao S., Xu J., Gao J., Xia Y. (2014). A 128-Stage Analog Accumulator for CMOS TDI Image Sensor. IEEE Trans. Circuits Syst. I..

[10] Snoeij M.F., Theuwissen A.J.P., Makinwa K.A.A., Huijsing J.H. (2006). A CMOS Imager with Column-Level ADC Using Dynamic Column Fixed-Pattern Noise Reduction. IEEE J. Solid-State Circuits.

[11] Sui X., Chen Q., Gu G. (2013). Adaptive grayscale adjustment-based stripe noise removal method of single image. Infrared Phys. Technol..

[12] Rogass C., Mielke C., Scheffler D., Boesche N.K., Lausch A., Lubitz C., Brell M., Spengler D., Eisele A., Segl K. (2014). Reduction of Uncorrelated Striping Noise–Applications for Hyperspectral Pushbroom Acquisitions. Remote Sens..

[13] Chang Y., Yan L., Fang H., Luo C. (2015). Anisotropic Spectral-Spatial Total Variation Model for Multispectral Remote Sensing Image Destriping. IEEE Trans. Image Process..

[14] Rakwatin P., Takeuchi W., Yasuoka Y. (2007). Stripe Noise Reduction in MODIS Data by Combining Histogram Matching With Facet Filter. IEEE Trans. Geosci. Remote Sens..

[15] Bouali M., Ladjal S. (2011). Toward Optimal Destriping of MODIS Data Using a Unidirectional Variational Model. IEEE Trans. Geosci. Remote Sens..

[16] Nie K., Li L., Yao S., Xu J. (2015). Scanning Modulation Transfer Function Model of TDI CMOS Image Sensor. J. Signal Process. Syst..

[17] Lepage G., Bogaerts J., Meynants G. (2009). Time-Delay-Integration Architectures in CMOS Image Sensors. IEEE Trans. Electron Devices.

[18] EMVA Standard 1288 release 3.1. http://www.emva.org/cms/upload/Standards/Stadard_1288/EMVA1288-3.1rc.pdf.

[19] Nakamura J. (2005). Image Sensors and Signal Processing for Digital Still Cameras.

